# Assessment of genetic variation in Bulgarian tomato (*Solanum lycopersicum* L.) genotypes, using fluorescent SSR genotyping platform

**DOI:** 10.1080/13102818.2014.901683

**Published:** 2014-06-04

**Authors:** Elena Todorovska, Albena Ivanova, Daniela Ganeva, Galina Pevicharova, Emil Molle, Bojin Bojinov, Mariana Radkova, Zhivko Danailov

**Affiliations:** ^a^ Agricultural Academy, AgroBioInstitute, Sofia, Bulgaria; ^b^ Agricultural Academy, Maritsa Vegetable Crops Research Institute, Plovdiv, Bulgaria; ^c^ Faculty of Ecology and Landscape Architecture, University of Forestry,Sofia, Bulgaria; ^d^ Faculty of Agronomy, Agricultural University,Plovdiv, Bulgaria; ^e^ Bulgarian Academy of Sciences, Institute of Plant Physiology and Genetics, Sofia, Bulgaria

**Keywords:** tomato (*Solanum lycopersicum* L.), SSR, genetic diversity, genetic distance, phylogenetic relationships

## Abstract

Genetic variability in modern crops is limited due to domestication and selection processes. Genetic variation in eight Bulgarian tomato varieties and breeding lines (variety Plovdivska karotina, variety IZK Alya, L21β, L53β, L1140, L1116, L975, L984) differing in their morphological and biochemical composition was assessed using a highly efficient and low-cost fluorescent simple sequence repeat (SSR) genotyping platform. Genotyping was conducted with 165 publicly available microsatellite markers developed from different research groups under a number of projects in tomato (SOL Genomics SSRs, Kazusa TGS and TES, SLM, TMS and LEMDDNa) among which only five (3.03%) failed to amplify the expected PCR fragments. Of the remaining markers, 81 (50.62%) were polymorphic in the whole collection of eight genotypes. Among the marker groups used, SLM markers were most polymorphic, followed by TMS and SOL Genomics SSR markers. The total number of amplified alleles was 299, with a mean of 1.869; and the average polymorphic information content (*PIC*) was 0.196. The genetic diversity within the collection was relatively low (0.2222). Nei's genetic distance varied from 0.0953 to 0.3992. Cluster analysis using the un-weighted pair group method with arithmetic mean (UPGMA) method indicated that the studied tomato genotypes are grouped in four main clusters, which is to some extent consistent with the morpho- and hemo-types of the studied tomatoes. Variety IZK Alya (cherry type) and two of the breeding lines (L1140, L1116) formed three separate and more distant clusters. The fourth cluster includes the other five genotypes. The observed grouping of these genotypes in two sub-clusters reflects their similar morphological and biochemical composition. The genetic distance information from this study might be useful for further implementation of breeding strategies and crosses among these inbred lines.

## Introduction

Tomato is one of the main vegetable crops all over the world, contributing both pro-vitamin A and vitamin C to the human diet and providing high economic value to producers and breeding industries in many countries. The number of accessions of cultivated and wild tomato species maintained in gene banks worldwide exceeds 62,000. The list of these gene banks includes the Asian Vegetable Research and Development Center in Tainan, Taiwan (http://www.avrdc.org), the Plant Genetic Resources Unit in New York, USA (http://www.usda.gov), the CM Rick Tomato Genetics Resource Center, The University of California, USA (http://tgrc.ucdavis.edu), and many others. The European Cooperative Program for Plant Genetic Resources (ECPGR) tomato database contains both molecular and phenotypic information of more than 20,000 accessions of several tomato species (http://documents.plant.wur.nl/cgn/pgr/tomato). The tomato core collection of the European *Solanaceae* database is composed of about 7000 domesticated (*S. lycopersicum* L.) lines, along with many representatives of wild species (www.eu-sol.wur.nl).

In addition to its worldwide agricultural and economic importance as a crop, tomato is a pre-eminent model system for genetic studies in plants.[1] As a model organism, tomato has been extensively used for both fundamental and applied studies in plant biology, with a focus on resistance to pests, plant development, and biochemical pathways.[2,3] For that reason, extensive genetic and genomic resources have been developed. The first high-resolution genetic map was constructed in the early 1990s, using more than 1000 restriction fragment length polymorphism (RFLP) markers between *Solanum lycopersicum* and a wild relative, *S. pennellii.*[4] Several molecular maps, based on crosses between the cultivated and wild species of tomato were further developed by Grandillo and Tanksley [5] in 1996, Bernacchi and Tanksley [6] in 1997, Chen and Foolad [7] in 1999, and Frary et al. [8] in 2004. The first plant resistance (R) gene isolated, cloned and characterized via a map-based cloning strategy was the tomato gene *Pto*, in 1993.[9] *Pto* confers resistance to the bacterium *Pseudomonas syringae*. Several other R genes were consequently isolated and characterized, e.g. *Cf-9*,[10] *Cf-2* [11] and *Ve1* [12] for resistance to fungal pathogens; *Mi* [13] for resistance to insects; and *Sw5* [14] and *Tm2*2 [15] for resistance to viral pathogens; as well as *sp* for growth habit [16] and *fw2.2*,[17] *ovate*,[18] and *sun* [19] for fruit development .

New resources for genetic analysis in tomato were provided in the last 10 years through genome sequencing projects. The results of large-scale sequencing of tomato expressed sequence tags (ESTs) reported in 2005 by Frary et al. [20] for example, identified 609 potential simple sequence repeats (SSRs) and 152 polymerase chain reaction-based (PCR-based) polymorphic markers that were mapped on the *S. lycopersicum* × *S. pennellii* reference population. Modern sequencing technologies, namely next-generation sequencing (NGS), provided a vast amount of information for sequence variation among individuals such as single nucleotide polymorphisms (SNPs), insertion/deletion variants (InDels) and copy number variations. In 2012, the recently developed “SolCAP” array including 7720 SNPs was used to generate high-density genetic maps, using two F_2_ interspecific populations: *S. lycopersicum* LA0925 × *S. pennellii* LA0714 (EXPEN 2000) population and Moneymaker × *S. pimpinellifolium* LA0121 (EXPIM 2012) population,[2] as well as to genotype SolCAP germplasm consisting of 426 accessions (410 inbreds and 16 hybrids) to study the effect of human selection on the tomato genome.[3] Such arrays give valuable SNP data for germplasm management in breeding programs and development of genomic selection strategies for crop improvement in tomato.[3]

In the course of domestication, as a result of intensive selection, the genetic diversity of cultivated tomato varieties has become narrower than in other crops. The selection of cultivated tomato (*Solanum lycopersicum* L.) has followed two main directions: (1) improvement of disease resistance and (2) fruit quality, assisted by introgression of genes from the wild *Lycopersicon* species. Nonetheless, tomato is known as a crop characterized by narrow genetic diversity. Less than 10% of the total genetic diversity in the *Lycopersicon* gene pool is found in *S. lycopersicum.*[21] This makes the identification of the best parental genotypes for crosses and the development of effective breeding strategies difficult. That is why the study of genetic diversity in elite tomato germplasm is one of the most important issues with an enormous impact on the effective management of genetic resources.

Currently, most of the molecular markers used for assessment of genetic diversity, inter-varietal differences and varietal identification in tomato, are PCR-based. These include RAPD (random amplified polymorphic DNA), SSR (simple sequence repeat, or microsatellite), ISSR (inter-simple sequence repeat), AFLP (amplified fragment length polymorphism), SCAR (sequence-characterized amplified region), CAPS (cleaved amplified polymorphic sequence), SNP and InDel markers. Among these markers, SSRs stand out as highly informative [22,23] and, unlike SNPs, do not require knowledge of the amplified sequences, further enzymatic manipulations,[24] or high-throughput technologies. The main advantage of SSRs is their co-dominant type of inheritance. With co-dominant markers, heterozygous individuals can be easily distinguished from homozygous, allowing the determination of allele configuration of each genotype and allele frequencies at different loci.[25] SSRs, however, require high-resolution fluorescent-based capillary systems to fully expand their informative potential.

In Bulgaria, the accessions of the cultivated and wild species of tomato are maintained in the gene banks of the Institute of Plant Genetic Resources (IPGR), Sadovo; the Institute of Vegetable Crops “IZK Maritsa”, Plovdiv; and the Institute of Plant Physiology and Genetics, BAS, Bulgaria. Most of the descriptions are based on pedigree information and morphological traits.[26–29] There is only one report on the use of ISSR markers for inter-varietal discrimination,[30] which speculates on the applicability of the system for studying the homogeneity of the material. It does not, however, provide sufficient information on the genetic diversity among tomato genotypes, necessary for the development of effective breeding strategies. Further assessment of elite germplasm at the molecular level is required.

In this study the genetic diversity in eight local tomato varieties and lines collected at IZK “Maritza” was assessed using fluorescence based SSR genotyping in order to reveal and explore the genetic variation available in tomato.

## Materials and methods

### Plant material

Eight Bulgarian accessions were studied: variety Plovdivska karotina of a *Solanum chillense* background, variety IZK Alya (cherry type) of a *Solanum pimpinellifolium* background and six tomato breeding lines (L21β, L53β, L1140, L1116, L975, L984) from the Maritza Institute of Vegetable Crops (Plovdiv, Bulgaria). Each genotype was presented by seven individual plants.

### DNA extraction, PCR procedure and SSR assay

#### DNA extraction

All DNA samples were extracted from frozen leaf tissue (250–300 mg) of field grown plants. A standard cethyl trimethyl ammonium bromide (CTAB) procedure was used,[31] with a modified extraction buffer containing 2% CTAB, 4% PvP, NaCl, Tris-HCl (pH 8.0) and EDTANa_2_ (pH 8.0). The addition of 8 mol/L of LiCl to the extraction buffer was a helpful step in the removal of the large amount of RNA at the initial step of the extraction. After several (three to four) chloroform–isoamyl alcochol extractions, DNA was precipitated with cold isopropanol. The DNA pellet was washed with 76% ethanol containing 10 mmol/L ammonium acetate for 30–45 min at 4 °C to eliminate the traces of polysaccharides. The dried DNA was dissolved in 1xTE buffer and the remaining content of RNA was removed by treatment with RNaseA for 45 min at 37 °C. After extraction with chloroform–isoamyl alcochol, DNA was precipitated with 5 mol/L NaCl to a final concentration of 0.2 mol/L and two volumes of 96% cold ethanol. DNA was further washed with cold 70% ethanol, dried and dissolved in a small volume of 1xTE buffer. The isolated DNA was of high quality (A260/280 = 1.7÷2.0). This allowed unproblematic amplification of microsatellite loci.

#### Primer synthesis

Published primers for tomato SSRs (http://solgenomics.net; http://marker.kazusa.or.jp/Tomato; Geethanjali et al. [32,33]; Areshchenkova and Ganal [34]; Smulders et al. [35]), referred to as locus-specific primers (LSPs), were synthesized with generic (M13) non-complementary nucleotide sequences *tagF* 5′ACGACGTTGTAAAA3′ and *tagR* 5′CATTAAGTTCCCATTA3′, respectively, at their 5′ ends as described by Hayden et al.[36] Primer aliquots (50 pM/μL) were prepared by mixing equimolar amounts of forward and reverse primers in miliQ H_2_O and were referred to as stocks primer sets for each locus. In addition, two generic *tag* primers, namely *tagF′* and *tagR*′, with the same sequences 5′ACGACGTTGTAAAA3′ and 5′CATTAAGTTCCCATTA3′ were also synthesized. The *tagF′* primer (5′ACGACGTTGTAAAA3′) was labelled at its 5′ end with one of the following fluorescent dyes: FAM, ATTO565, ATTO550 and YAKIMA YELLOW (Applied Biosystems), allowing direct detection of alleles on an automated capillary sequencer (ABI3730, Applied Biosystems). All primers were synthesized by Microsynth.

#### PCR assay

All uniplex PCR reactions were performed according to Hayden et al. [36] in a 6 μL reaction mixture containing 20–25 ng of genomic DNA, 2× MyTaq HS mix (Bioline), 75 nmol/L of each dye-labelled *tagF′* and unlabelled *tagR*′ primers, and an appropriate concentration (20, 30 or 60 nmol/L) of unlabelled LSP, depending on the marker groups used.

PCR was performed on Veriti96 Thermal Cycler (Applied Biosystems) using the following PCR conditions, depending on the melting temperature of the locus-specific primers. These were performed according to Hayden et al. [36] and Tsonev et al. [37] with some minor modifications:

1. *PCR program 50 °С* included a denaturing step at 95 °С for 3 min, followed by 10 cycles of amplification, each including a denaturing step at 92 °С for 30 s, an annealing step at 50 °С for 1.30 min, and a synthesis step at 72 °С for 1 min; 20 cycles: 92 °С for 30 s, 63 °С for 1.30 min, 72 °С for 1 min; 40 cycles: 92 °С for 15 s, 54 °С for 30 s, 72 °С for 1 min; and final extension at 72 °С for 10 min.
2. *PCR program 63 °С* included a denaturing step at 95 °С for 3 min, followed by 25 cycles of amplification each including a denaturing step at 92 °С for 30 s, 63 °С for 1.30 min and a synthesis step at 72 °С for 1 min; 40 cycles: 92 °С for 15 s, 54 °С for 30 s, 72 °С for 1 min; and final extension at 72 °С for 10 min.

### SSR analysis

Electrophoresis and visualization of tomato SSRs was performed on an ABI3730 DNA analyser (Applied Biosystems). A standardized multi-pooling procedure was used to prepare SSR products for electrophoresis. The post-PCR mixing of the amplified products was performed as follows: (1) each product was diluted by addition of two volumes of miliQ H_2_O (1:2); (2) the diluted samples were pooled together at a ratio of 1:1:1:1 for FAM:ATTO565:ATTO550:YAKIMA YELLOW (FAM:PET:NED:VIC) to give a final volume of 25 μL (1:75 final dilution of each PCR product) or 20 μL (1:60 final dilution); (3) 3 μL of the diluted pooled samples were mixed with 8 μL of deionized formamide containing 0.1 μL of GelScan500 LIZ size standard, denatured for 3 min at 92 °С and electrophoresed on an ABI3730 DNA analyser. SSR allele sizing was performed with Gene Mapper v.4 software (Applied Biosystems).

The pooling of PCR products with different dye-labels at 1:1:1:1 aimed to account for differences in the relative fluorescence of each fluorophore. In cases where the intensity of SSR bands specific for a particular SSR was two to three times higher, the ratio was changed to roughly equalize the intensity of bands labeled with different fluorophores.

### Biochemical analysis

The content of lycopene (mg per 100 g) and β-carotene (mg per 100 g) was determined according to Manuelyan.[38]

### Data analysis

Amplified fragments were scored for the presence (1) or absence (0) of the respective bands in all the genotypes tested.

The sample allele frequencies were calculated as *p_u_* = *n_u_*/(2*n*), where *n* is the number of individuals.

The genetic diversity index was calculated for each primer and each pattern frequency: *H* = 1 − Σ*Pi2*, where *H* is the genetic diversity index and *Pi* is the pattern's frequency.[39]

The polymorphism information content (*PIC*) [40] for each SSR (marker polymorphism) was calculated according to the formula: *PIC* = 1 − Σ*pi*2, where *pi* is the frequency of the *i* ^th^ allele for each SSR marker locus in the set of eight tomato varieties and lines investigated.

Heterozygosity is simply the proportion of heterozygous individuals in the population. At a single locus it is estimated as

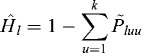



The Nei distance matrix was used to construct the dendrogram with the un-weighted pair group method with arithmetic mean (UPGMA) module of Power Marker 3.25.[41]

## Results and discussion

Tomato is one of the many autogamous crop species. Its germplasm diversity has been reduced several times by the process of domestication and, further, by breeding new cultivars outside the region of origin. This makes identification of polymorphic markers in elite tomato germplasm difficult. Due to the lack of genetic markers that reveal differences between elite tomato breeding lines, the comprehensive study of most economically important traits within genetic backgrounds relevant to plant breeding has been hindered.

Different molecular markers, including RFLP, RAPD, AFLP, ISSR and SSR have been widely used to study genetic diversity in tomato.[21,42–47] However, most of these markers identify limited polymorphisms in cultivated tomatoes.[20,21,47] In addition, some types of markers (e.g. AFLP and SSR) are clustered in certain chromosomal regions.[48] Therefore, they are not so useful in tomato genetic studies and breeding.

Recently, the collection of PCR-based markers was enlarged by addition of new SSRs [32,33,49] developed from expressed-sequence tags (TES markers), genomic sequences (TGS markers) and from anchored BAC clones (SLM markers). The large number of SSR markers developed until now, provides an easily handling genome tool for molecular breeding in tomato. Although great morphological variations have been observed in tomato,[50] the genetic variation within tomato cultivars is approximately 5%.[21]

In our study, we used eight tomato varieties and lines with large variation in fruit characters, including size (large, medium, small), shape (round, flat round, oval), colour (yellow–orange, red–orange, red, red–violet, pink, purple–black), number of fruit locules, etc., as well as growth habit (determinate and indeterminate). Some variations were also observed in fruit quality characters (Table 1). The lycopene and carotene content varied from 0.65 to 9.23 and from 0 to 4.76, respectively.[28,29] However, high morphological variability is not always reflected at the molecular level,[51] and detailed molecular genetic studies are required when crosses between elite germplasm are planned.

**Table 1.** Phenotypic and fruit quality traits of Bulgarian tomato varieties and breeding lines.

						Lycopene (mg per 100 g)		β-carotene (mg per 100 g)	
Variety/breeding lines	Growth habit	Shape	Inflorescence type	Number of fruit locules	Fruit colour	± sd	CV (%)	± sd	CV (%)
Plovdivska karotina	Indeterminate	Flat round	Simple	2–4	Red–orange	4.17 ± 1.04	24.86	4.76 ± 0.82	17.25
L21β	Indeterminate	Round	Simple	5–7	Yellow–orange	–	–	4.46 ± 0.92	20.59
L1116	Indeterminate	Flat round	Simple	5–7	Red–violet	6.15 ± 1.36	22.12	0.28 ± 0.19	68.49
L1140	Indeterminate	Round	Simple/complex	2–4	Purple–black	2.80 ± 0.45	16.01	2.12 ± 0.62	29.05
IZK Alya	Indeterminate	Oval	Simple	2–3	Red	9.23 ± 1.18	12.81	–	–
L984	Determinate	Oval	Simple	2–3	Red	8.64 ± 1.16	13.45	–	–
L975	Indeterminate	Oval	Simple	2–3	Pink	7.08 ± 0.69	9.73	–	–
L53β	Determinate	Flat round	Simple	5–7	Orange	0.65 ± 0.10	14.87	2.78 ± 0.34	12.28

Note: CV: coefficient of variation.

### Characteristics of SSR markers

A total of 165 publicly available tomato microsatellite markers were used to assess the genetic diversity in a set of eight Bulgarian inbred lines and varieties. Among them, 100 were genomic- and EST-SSR markers from the SOL Genomics Network (http://solgenomics.net); 32 were TGS and TES markers from the Kazusa Tomato Genomics Database (http://marker.kazusa.or.jp/Tomato); 16 were SLM markers developed from anchored BAC clones of chromosomes 6 and 12 [32,33]; 16 were TMS and EST markers developed by Areshchenkova and Ganal [34] and one SSR-LEMDDNa of Smulders et al.[35] The markers were selected to cover almost all 12 chromosomes with a minimum of seven SSRs per chromosome and to be located near to already published QTLs and genes responsible for phenotypic variation in quality and other morpho-physiological traits.

In this study, standardized PCR conditions at two different temperatures of annealing (50 and 63 °С) were employed (Table 1S in the Online Supplementary Appendix), which enables further amplification of markers to be deployed for multiplexed amplification.

**Figure 1.**
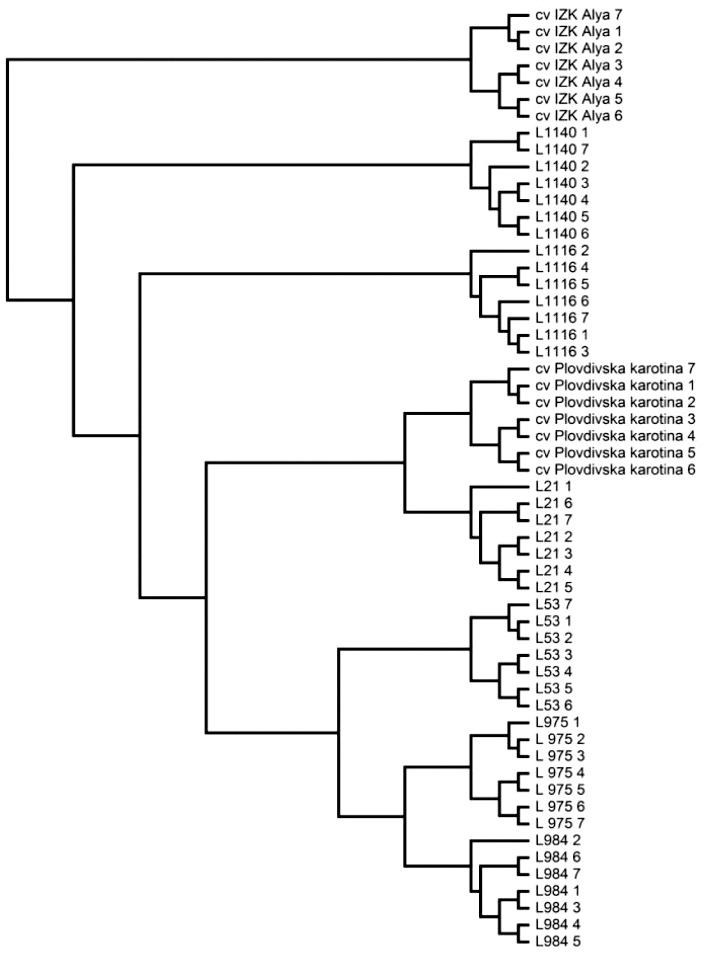
Dendrogram based on Nei's [39] genetic distance, summarizing the data on differentiation among eight varieties and lines, according to SSR analysis.

Standardized PCR conditions were achieved by performing an initial optimization step for each locus-specific primer concentration (20, 30, 50, 60 and 80 nmol/L). Adjusting the locus-specific primer concentration enables the PCR specificity and yield to be controlled and helps to prevent the non-specific annealing of locus-specific primers during the first few PCR cycles.[36] Our test showed that primers for all SOL Genomics SSRs, TES, TGS, TMS, EST and LEMDDNa markers gave clear amplification products at a concentration of 20–30 nmol/L while SLM primers showed the best amplification at a concentration of 60 nmol/L. Table 1S (Online Supplementary Appendix) demonstrates that all SOL Genomics and SLM SSRs, some of the TGS, TES and most of the TMS and EST primers amplified products at 50 °С. This optimization step was a prerequisite for correct and efficient amplification of microsatellite alleles in all tested genotypes (Fig. 1S in the Online Supplementary Appendix) and eliminated the need to use complex touchdown PCR or to adjust the annealing temperatures for each locus. Thus, we were able to amplify loci for which annealing temperatures below 50 °C are reported.

In our study, only five out of 165 primers (3.03%) failed to amplify the expected PCR fragments. Seventy-nine (49.38%) markers amplified monomorphic banding patterns while the other 81 markers (50.62%) generated polymorphic ones (Table 2). These results show that only a few SSR loci were not amplified with the new fluorescent-based SSR genotyping used by us, which was developed following the protocol of Hayden et al. [36] and Tsonev et al. [37] In other studies this percentage is much higher. For example, Benor et al. [52] reported that 31.7% of the 60 SSRs used failed to amplify the expected PCR products while El-Awady et al. [1] scored a total of 10% SSRs that were not able to produce amplicons.

**Table 2.** Total number of polymorphic loci detected with different marker groups.

Marker groups	Number of microsatellite loci	Number of polymorphic loci	Polymorphic loci (%)
TMS and EST	15	10	66.66
SSR	99	50	50.51
SLM	15	15	100.00
TGS and TES	30	5	16.66
LEMDDNa	1	1	100.00
Total	160	81	50.62

A total of 299 alleles were detected at the 160 SSR loci in our study. The mean *PIC* for all 160 SSR markers was 0.196 with values ranging from 0.00 for markers generating monomorphic bands, to 0.786 for the marker SLM6-7. The number of alleles per locus varied from 1 to 6 with a mean of 1.869 alleles per locus (Table 3).

**Table 3.** Number of amplified microsatellite loci, total and mean number of alleles, gene diversity (*GD*), observed heterozygosity (*Ho*) and polymorphic information content (*PIC*).

	Number of amplified loci	Number of alleles	*GD*	*Ho*	*PIC*
Total	160	299	35.553	6.625	31.439
Mean		1.869	0.222	0.041	0.196

These results indicated that allelic variation in the studied tomato varieties and lines is limited. Benor et al. [52] reported 4.3 alleles per locus on average after testing of polymorphic loci only (35 out of 41 amplified) in 39 determinant and indeterminant tomato inbred lines selected from China, Korea, Japan and the USA, and a *PIC* value of 0.31. Smulders et al. [35] detected three alleles per locus on average, after testing of 30 SSR loci on seven inbred lines of tomato. He et al. [53] identified 2.7 alleles per locus on average and a *PIC* value of 0.37 in a study of relationships among 17 varieties and two parental lines of tomato with 60 SSR markers. Limited allelic variation was also observed in a study of tomato populations consisting of a total of 216 genotypes from four breeding centres in China, using 12 SSRs and 35 SNP markers.[54]

In our study, the majority of polymorphic SSR loci generated two (55.55%) and three (29.63%) alleles, followed by four alleles (12.35%). Most of the SSR loci for these tomato samples contained di-nucleotide (47%), tri-nucleotide (28.4%) and complex repeats (20.9%) and only 1.2% of them had tetra-nucleotide repeats (Table 1S in the Online Supplementary Appendix). AT and TA were the most common repeat types (22.22% and 17.28%, respectively), followed by CGG, TTC and AAG (3.7% each). Similar results have been reported by Benor et al. [52] for 35 polymorphic SSR markers. There are several reports that the allelic variation might correlate with the number of repeats within a particular locus. A positive relationship has been found between the number of repeats and *PIC* in earlier reports in tomato.[35,53,55] However, no such relationship was found in the present investigation. In our study, SSR63 with the lower *PIC* value (0.629) has 39 AT repeats, as compared to SLM6-7 with 22 repeats and a *PIC* value of 0.786. Unlike the reports of He et al. [53] and Benor et al. [52] who found no relationship between *PIC* and the number of nucleotides per repeat, our study is in agreement with those of Blair et al. [56] and Jones et al.[57] Both authors observed that the polymorphism level in tri-nucleotide repeats is lower than that in di-nucleotide repeats in rice and ryegrass.

The SSR marker groups used here showed different level of polymorphisms in the eight studied Bulgarian tomato genotypes (Table 2). The highest level of polymorphism (100%) was generated with SLM markers developed from anchored BAC clones of chromosomes 6 and 12.[32,33] All 15 amplified SLM loci were polymorphic. Among the employed 99 genomic- and EST-SSR markers from the SOL Genomics Database (http://solgenomics.net), 50.51% were polymorphic while TMS and EST-SSR markers of Areshchenkova and Ganal [34] showed 66.66% polymorphism. The lowest level of polymorphism (16.66%) was observed within TGS and TES loci (http://marker.kazusa.or.jp/Tomato), which are expected to be more appropriate for distinguishing of elite tomato germplasm and its wild relatives or landraces. Since some of the markers used here are located near to QTLs for quality traits and genes for some morpho-physiological characteristics,[44] their further use in association studies is highly recommended.

### Genetic diversity levels

The mean genetic diversity over 160 microsatellite loci in the studied set of eight Bulgarian varieties and lines was found to be relatively low (*GD* = 0.2222, or 22.22%). Similar results were observed in the study of Chen et al.,[54] who reported an overall genetic variation of 19.16% across 47 SSR and SNP loci in 216 cultivars, hybrids and elite breeding lines originating from four breeding centres in China. Using the UPGMA clustering approach, Benor et al. [52] observed a separation of the inbred lines into four groups at a genetic similarity value of 0.85, which is also an evidence for a low level of genetic diversity in the tomato germplasm studied. However, most of the investigations on genetic diversity in tomato are based on a preliminarily selected set of highly polymorphic markers. After removal of monomorphic loci (comprising about 49% of the total analysed loci in our study) the level of the *GD* increases twofold (to 0.41) (data not shown). However, this value does not reflect the proper genetic variation in tomato and, therefore, a caution should be taken when planning breeding strategies.

To examine the genetic relationships based on SSR results among the eight Bulgarian tomato varieties studied, the data scored from all 160 markers were compiled and analysed according to Nei.[39] The genetic dissimilarity matrices are shown in Table 4.

**Table 4.** Pair-wise genetic distances in different tomato varieties.

	IZK Alya	L975	L1116	L1140	L21β	L53β	L984	Plovdivska karotina
IZK Alya	0.0000							
L 975	0.3688	0.0000						
L1116	0.3754	0.2134	0.0000					
L1140	0.3053	0.2317	0.2660	0.0000				
L21β	0.3235	0.1610	0.1708	0.2569	0.0000			
L53β	0.3500	0.1625	0.2120	0.2460	0.1672	0.0000		
L984	0.3992	0.1376	0.2086	0.2934	0.1352	0.1439	0.0000	
Plovdivska karotina	0.3688	0.1813	0.1544	0.2585	0.0953	0.1625	0.1546	0.0000

The largest genetic distance was observed between IZK Alya and breeding line L984 (0.3992) and between IZK Alya and L1116 (0.3754) while the lowest one was detected between variety Plovdivska karotina and line L21β (0.0953). The latter genotypes are indeterminant and have a similar content of beta-carotene and fruit colour (Table 1). Low genetic distance was also observed between L21β and L984 (0.1352), and between L975 and L984 (0.1376). The first pair of cultivars is characterized by a similar fruit shape, brix and titratable organic acids content (data not shown), while the second one includes genotypes with a common origin and similar morphological and biochemical composition (Table 1).

IZK Alya is the most diverse variety in comparison to the other genotypes. It is a cherry-type tomato (with *Solanum pimpinellifolium* germplasm in its background) and is characterized by the highest content of lycopene (9.23 ± 1.18) (Table 1), ascorbic acid, total pigments and titratable organic acids (data not shown). Twenty unique SSR alleles, not found in the other genotypes of the collection studied here were detected in IZK Alya. These can be used for discrimination and protection purposes in the frame of this collection.

The distance matrix based on SSR data was used to construct a dendrogram (Figure 1). The dendrogram is divided into four main clusters. The first one includes variety IZK Alya. The second one includes breeding line L1140, and the third main cluster contains L1116. The fourth cluster is separated into two sub-clusters, one including the closely related genotypes (variety Plovdivska karotina and L21β), and the second one including L53β, L975 and L984. Lines L975 and L984 are more narrowly related than to L53β, basically due to their similar fruit shape, brix, titratable organic and lycopene content, and common origin.

In addition, the dendrogram clearly showed the precise grouping of the seven studied DNA samples per genotype. Even though the average level of heterozigosity was low (*Ho* = 0.0414), few genotypes (L1140, L1140 and L984) are characterized by a higher level of variability due to heterogeneity and/or heterozigosity in some loci such as SSR95 (1 chr.), SSR96 (2 chr.), LEMDDNa (5 chr.), SSR350 (5 chr.), SSR276 (7 chr.), SSR344 (8 chr.), SSR70 (9 chr.), SLM12-12 (12 chr.) and TMS33 (12 chr.).

The data reported here are in support of the fact that SSR markers are an appropriate tool for performing unambiguous cultivar discrimination and determining of genetic heterogeneity in tomato populations. The latter is probably the main case for the difficulty in discriminating material from hybrids when analyses are carried out exclusively based on compositional parameters.[58]

## Conclusions

The results of this study confirm the efficiency of the employed fluorescent SSR genotyping platform in assessing and discriminating local tomato genotypes. It may be further improved through conversion of uniplex into multiplex PCR, appropriate for application in projects related to mapping and association studies in tomato.

The study showed a low level of genetic diversity in the studied collection of eight Bulgarian tomato genotypes, basically due to specific selection strategies aiming at early ripening, high and stable yield, resistance to biotic and abiotic stress as well as fruit quality. Although the major cultivated local tomato genotypes in Bulgaria are well described at the phenotypic and biochemical level, further molecular studies are necessary in order to determine the precise genetic structure of the local varieties, populations, landraces, hybrids, introduced accessions and wild species deposited in gene banks in Bulgaria. Such studies should be useful both for identification of duplicate accessions and establishment of a core collection in the gene banks, as well as for sustainable conservation of the genotypes collected. Precise molecular characterization of collections will allow more efficient management and utilization of genotypes in breeding programs.
